# Northwestern Pacific tropical cyclone activity enhanced by increased Asian dust emissions during the Little Ice Age

**DOI:** 10.1038/s41467-022-29386-2

**Published:** 2022-03-31

**Authors:** Yang Yang, David J. W. Piper, Min Xu, Jianhua Gao, Jianjun Jia, Alexandre Normandeau, Dongdong Chu, Liang Zhou, Ya Ping Wang, Shu Gao

**Affiliations:** 1 School of Marine Science and Engineering, Nanjing Normal University, Nanjing, 210046 China; 2 Natural Resources Canada, Geological Survey of Canada (Atlantic), Bedford Institute of Oceanography, Dartmouth, NS B2Y 4A2 Canada; 3 School of Geography and Ocean Science, Ministry of Education Key Laboratory for Coast and Island Development, Nanjing University, Nanjing, 210093 China; 4 State Key Laboratory for Estuarine and Coastal Research, School of Marine Sciences, East China Normal University, Shanghai, 200062 China; 5 Institute of Physical Oceanography and Remote Sensing, Ocean College, Zhejiang University, Zhoushan, 316000 China; 6 School of Geography, Geomatics and Planning, Jiangsu Normal University, Xuzhou, 221116 China

**Keywords:** Palaeoceanography, Palaeoclimate

## Abstract

Instrumental records reveal that intense tropical cyclone (TC) activity varies with tropical sea surface temperature (SST) on annual-decadal scales. Drivers of intense TC activity at the centennial-millennial scale are less clear, due to the sparseness of pre-observational reconstructions. Here, we present a new 2 kyr continuous activity record of intense TCs from offshore eastern China. Our reconstruction indicates that this site witnessed enhanced TC activity during relatively warm periods, with a widespread increase in TC activity during the later part of the Little Ice Age. This latter observation reveals that enhanced TC activity was synchronized with increased Asian dust emissions during the Little Ice Age. TC activity was also lower in the late Roman Warm Period, when SST was higher but Asian dust emissions were lower than in the early phase. Such patterns suggest a centennial-millennial link between TC climatology and a combination of SST changes and Asian dust levels.

## Abstract (web summary)

Here, the authors show that enhanced tropical cyclone activity was synchronized with increased Asian dust emissions during the Little Ice Age, implying a mechanism linking tropical cyclone climatology to Asian dust levels, at 100 to 1000 year scales.

## Introduction

Tropical cyclones (TCs) and the resultant storm surges, high wind speeds, giant waves, and heavy rainfall have devastating socioeconomic impacts around the world^1,2^. Climate controls the characteristics of TCs by providing environmental conditions that affect their formation, intensity, and movement^3–5^. Therefore, resolving how TC activity may respond to future climate warming is attracting increasing research attention^6–8^. Both instrument-based observations^1,9^ and model projections^10,11^ indicate that global warming may enhance the intensity and frequency of intense TCs (i.e., categories 4 and 5). However, due to the short observational record, it is uncertain whether significant recent changes in TC activity have been identified in instrumental observations^12,13^ and whether any increase was caused by natural changes^14,15^ or anthropogenic forcing^9,16^. Understanding the mechanisms by which TC activity has varied in response to past forcing may help elucidate the drivers of TC variability under future warming scenarios.

Coastal sedimentary archives provide a means to extend our knowledge of TC dynamics and associated climate forcing beyond instrumental records, and potentially provide an opportunity to test the hypothesis that the intensity and frequency of intense TCs have increased due to global warming as a consequence of increases in sea surface temperature (SST). In the last decades, there have been a plethora of TC reconstructions seeking to establish a longer time perspective on recent TC activity^17–20^. These reconstructions focused mostly on the long-term frequency pattern of TC activity, showing alternating periods (centuries to millennia) of relative quiescence and of heightened TC activity^19–22^. However, the dearth of TC reconstructions spanning the observation period hinders the assessment of the role of TC frequency and intensity in creating event layers^23–25^. Thus, the identification of climate patterns influencing TC frequency and intensity has been poorly constrained. It has been suggested that periods of increased TC activity correlate to the occasions of warmer SSTs on long-term temporal scales^21,25,26^, but there is an alternative view that increased TC activity also occurred during colder times^27,28^, e.g., the Little Ice Age^29,30^. To date, the centennial- and millennial-scale variability of past intense TC activity and its response to temperature changes remain unclear, particularly for the northwestern Pacific region. In addition, an inverse correlation between African dust and Atlantic TC frequency has been documented over the past 50 years (i.e., 1985–2005 CE)^31,32^. Such a correlation is not stationary in time due to microphysical–radiative interactions of aerosols^33^. Sedimentary reconstructions of African dust and storm activity suggest that the substantial increase in African dust over the last 200 years and in the early Holocene are coeval with higher Atlantic TC activity^33^. However, the influence of Asian dust emissions on TC activity in the northwestern Pacific has not yet been explored on timescales greater than annual-decadal.

**Fig. 1:**
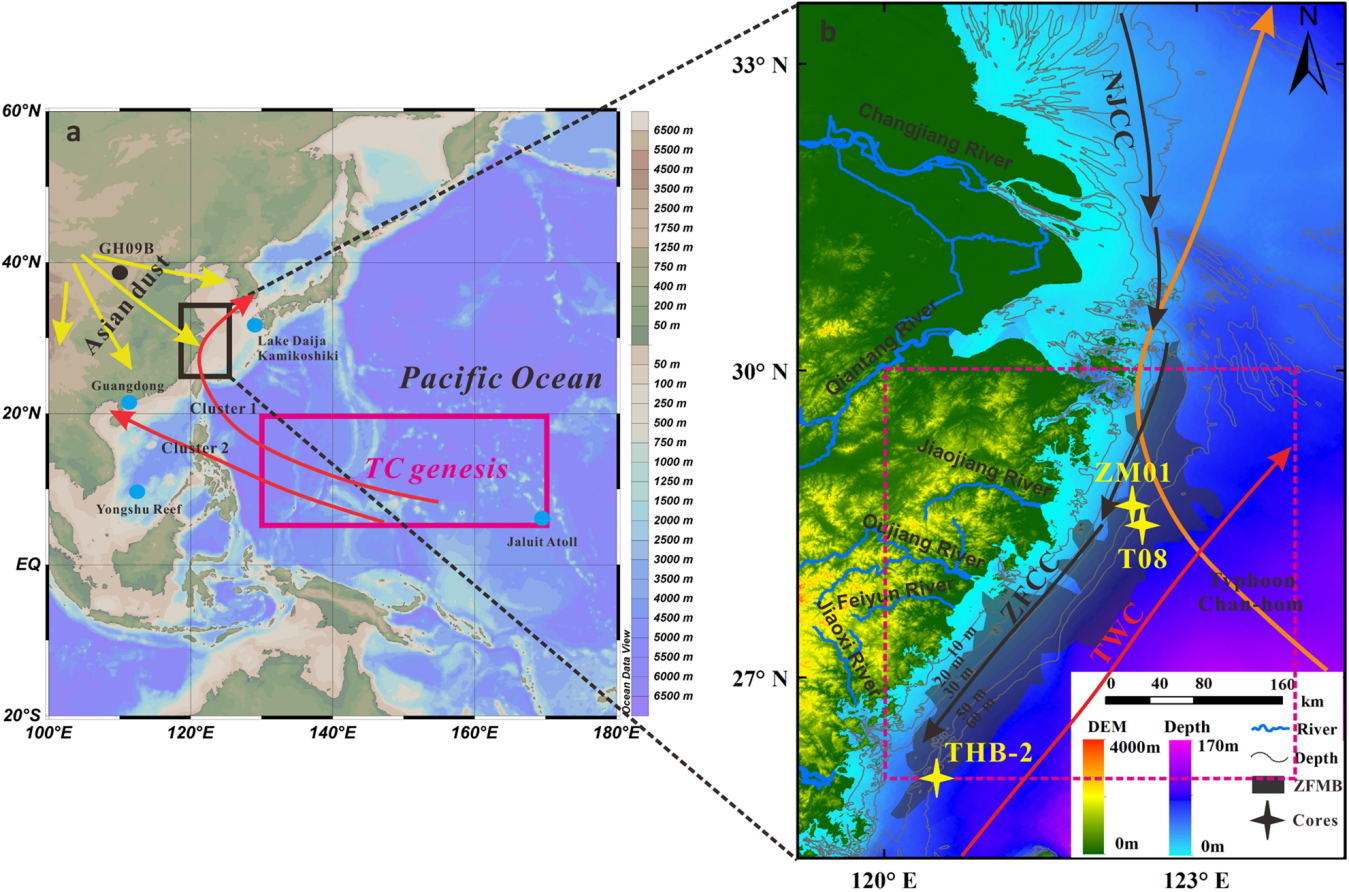
Site location. **a** Overview map of the northwestern Pacific Ocean showing the region of TC genesis (red rectangle), main TC tracks (red arrows), published paleostorm records^18, 27, 30, 54, 55^ (blue dots) and the schematic trajectories of Asian dust storms (yellow arrows). The location of the Asian dust proxy site from Lake Gonghai, northern China^66^ (GH09B) used in this study is also indicated (black dot). b Locations of cores ZM01, T08^25^, and THB-2^38^ (yellow stars). A simplified illustration of the Zhejiang-Fujian mud belt (gray area), instrumental wind speed area (red dashed box), and the track of 2015 CE Super Typhoon Chan-hom (orange arrow) are also shown. ZFCC Zhejiang-Fujian Coastal Current (black arrow), TWC Taiwan Warm Current (red arrow), NJCC Northern Jiangsu Coastal Current (black arrow).

The late-Holocene includes the most recent warm and cold periods on a geological timescale with a series of abrupt climatic events, including the Roman Warm Period (RWP, ~250 BCE–450 CE), the Dark Ages Cold Period (DACP, ~450–800 CE), the Medieval Warm Period (MWP, ~800–1250 CE), the Little Ice Age (LIA, ~1400–1850 CE), and the Current Warm Period (CWP; ~since 1950 CE)^34–37^. Records from this time interval enable us to quantify the relationships between TC activity and changes in SST and Asian dust emissions. Fortunately, in Chinese coastal seas, there are large amounts of muddy sediments that are rapidly and continuously deposited in low-energy environments (e.g., Zhejiang-Fujian inner-shelf mud belt, eastern China). They have been widely used for paleoenvironmental studies^38,39^, and therefore can provide high-resolution records of past changes in TC activity, as paths of many northwestern Pacific TCs intersect the coastlines of Zhejiang and Fujian provinces, e.g., 2015 CE Super Typhoon Chan-hom (Fig. 1).

In this study, we undertake such an exercise, using a simple approach of linking sediment grain size to instrumental records of TC-induced wind speed. The results have a sufficiently high correlation with the observed TC-induced wind speed to justify extracting an estimate of the magnitude of intense TC activity over the last 2000 years. Reconstructions of paleo TC activity from sedimentological proxies were supplemented by numerical modeling of the hydrodynamic conditions of the bottom boundary layer during fair weather and storm events. Combined with existing paleoclimate records, we then explore the associations between climatic forcings and TC activity during the recent warm and cold periods.

## Results

### Lithology and chronology

Core ZM01, located in the Zhejiang-Fujian mud belt (ZFMB), offshore eastern China, is mainly composed of silt and clay, accounting for 84.7 and 14.4% on average, respectively (Fig. S1). The proportion of sand fraction varies from 0 to 7.7%, with an average of 0.9%. The illite/smectite and kaolinite/chlorite ratios are relatively steady (Fig. S1) and represent a constant deposition of clay minerals, indicating a stable sediment supply. The chronology was established using ^210^Pb-^137^Cs dating of bulk sediments and ^14^C dating of benthic foraminifera from core ZM01 (Fig. S2 and Table S1). The results indicate that the upper 192 cm of the core dates between approximately 39 CE and 2018 CE, and the temporal resolution at 1 cm spacing of grain-size analyses is about 10 years. The sedimentation rates for the upper (0–40 cm) and lower (40–192 cm) parts of the core are ~12.4 mm/year and 0.80 mm/year, respectively, which are consistent with reported sedimentation rates over the region^25,38^.

### TC indicator

**Fig. 2:**
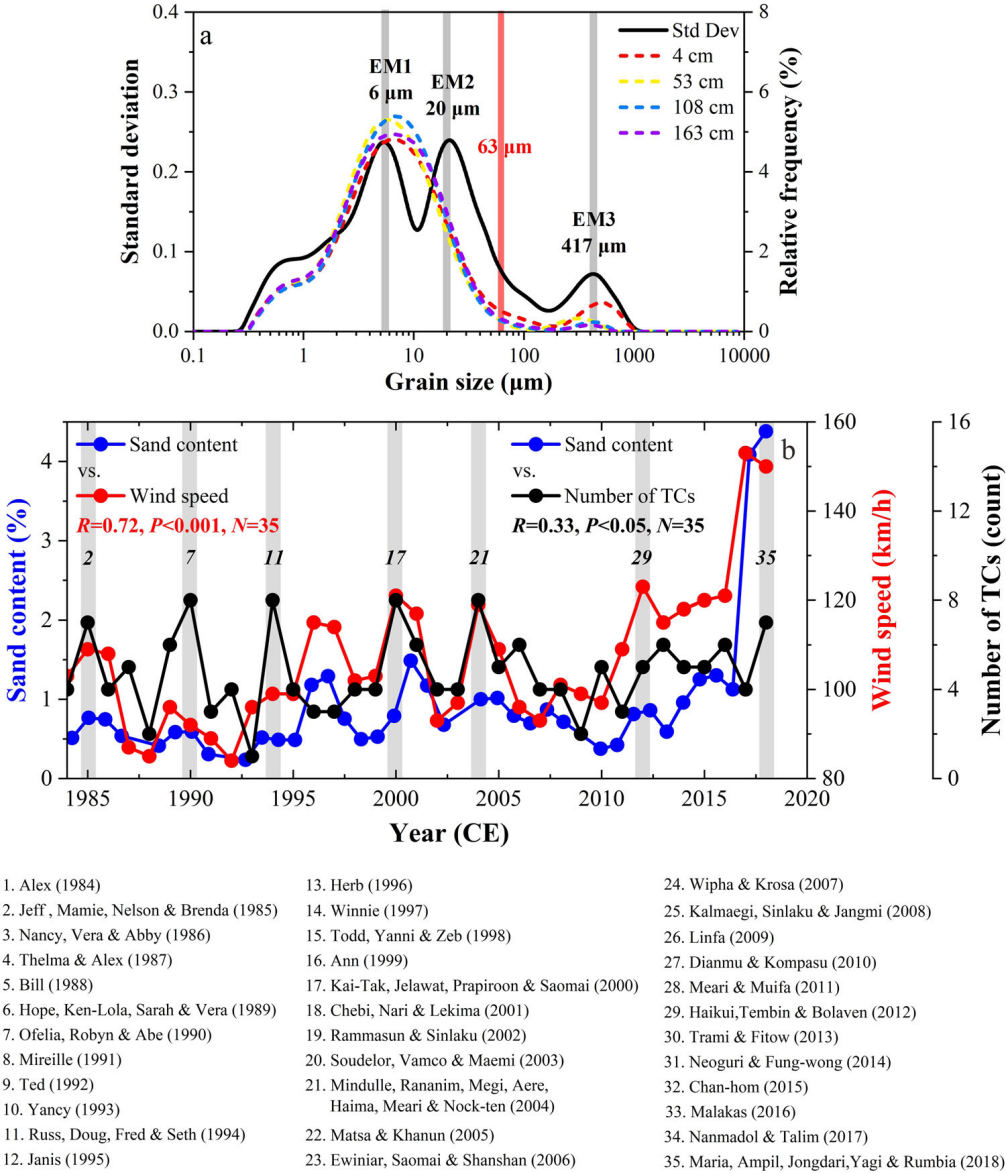
Establishment of a TC activity proxy in the sediments of the ZFMB. **a** Results of grain-size analysis using grain size-standard deviation approach. Black line is a plot of standard deviation against grain size. Colored dotted lines show the grain-size distributions of selected samples. **b** Comparison of the sand content of core ZM01 with the number of TC events and the annual maximum wind speed of TCs passing through the region (1984–2018 CE). Selected TC events are numbered 1–35.

Core ZM01 was used to explore TC dynamics over eastern China, focusing on the past ~2000 years because the depositional environment has been stable since that time^25,40^. Three grain-size components were separated from the grain-size distribution curves (Fig. 2a): the very fine silt component (EM1) with a peak of ~6 μm, the medium silt (EM2) with a peak of ~20 μm, and the medium sand (EM3) with a peak of ~417 μm. The fine-grained components (EM1 and EM2) represent the normal deposits of the ZFMB transported by the coastal currents driven by the East Asian Winter Monsoon^39^ (EAWM). Model simulations suggest that coastal currents have insufficient energy to transport coarse materials^41^ (i.e., EM3), and an EAWM record related to ZFCC is always reconstructed from the fine-grained fractions of <45 μm^39^. Further, the grain‐size distribution of sediments substantially affected by coastal currents is usually unimodal, but that affected by both storms and coastal currents is bimodal, with an additional coarse-grained component^39^ (Fig. 2a).

Event beds contained in the coastal sedimentary record may be associated with a variety of extreme events, including typhoons, river floods, or tsunamis^25,42,43^. Core ZM01 is far away from the Changjiang estuary, and variations in the sand content does not exactly match the record of major floods^44^ (Fig. S3), which is controlled by a combination of the East Asian Summer Monson (EASM) and/or TC activity. The coarse material supplied to core ZM01 is unlikely to be transported directly by fluvial discharge. Likewise, the East China Sea is little affected by tsunamis due to the shallow-water damping of tsunami waves, according to numerical simulations of the region^42,45^. However, tsunamis in Japan or the South China Sea may have a remote influence on the sedimentary record of the East China Sea. For example, the most significant change in the grain size sequence of the <58 μm fraction in the ZFMB is temporally consistent with the tsunami in the South China Sea^42^ (e.g., the 1024 CE South China Sea tsunami). Such remote tsunami effects on the sedimentary record of the East China Sea are likely to be concentrated in fine-grained sediment fractions smaller than 63 μm. Thus, the sand content variations near our site are unlikely to have been caused by river floods or tsunamis. Using the boundary between silt and sand (i.e., 63 µm), we define the coarse-grained fraction of >63 μm (i.e., sand content) as a proxy of intense TC activity for the ZFMB region.

Both TC intensity and frequency affect the sand content, so this proxy in sedimentary records potentially contain information on both parameters. The regression of the sand content in core ZM01 against the instrumental annual maximum wind speed of TCs passing through the region yields a significant and positive correlation (*R* = 0.71, *P* < 0.001, *N* = 35) over the past 35 years (1984–2018 CE) (Fig. 2b), with greater sand content corresponding to higher TC intensity. Elsewhere, the content of coarse-grained fraction in sediments of the ZFMB has been found to link to TC frequency^25^. Comparing the sand content with the instrumental records of TC frequency (Fig. 2b) also shows a positive correlation (*R* = 0.35, *P* < 0.05, *N* = 35), suggesting that, to some extent, TC frequency is a forcing mechanism of the sand content changes near our study site. The annual average maximum wind speed of TCs around core ZM01 is about 107 km/h (Fig. 2b), indicating that intense TCs near our site are likely to be consistent with category 1 intensity.

Sediment particle size is generally a local expression of TC intensity/frequency and is mainly related to variables such as storm proximity, storm translation speed, angle of approach, and storm wind speed^46–48^. Four clusters of TCs in the northwestern Pacific have been identified with different geographic locations of genesis and tracks^49^. Clusters 1 and 2 include the most numerous TCs (Fig. 1), accounting for around 34 and 30% of the TCs, respectively. TCs in Clusters 1 and 2 travel northwest-to-northwards and west-to-northwestwards, respectively, and make landfall mainly over East Asia and Southeast Asia. TCs in Clusters 3 and 4 are generated farther east than those in the first two groups, moving from northwest to northeast, and generally stay over the open ocean during their lifetime, with a much smaller chance of making landfall (Fig. S4). The core site of the ZFMB is located in the transition zone between the first two groups, and is mainly influenced by TCs of Cluster 1 and partially by Cluster 2. Due to the large number and high intensity of TCs in Cluster 1^49^, the peak intensity of their TCs, to some extent, reflects the variation of the average peak intensity of TCs in the northwestern Pacific. The regression of the annual maximum wind speed of TCs affecting the Zhejiang coast against the annual mean TC peak intensity over the northwestern Pacific yields a significant and positive correlation (*R* = 0.53, *P* < 0.001, *N* = 35) for the past 35 years (Fig. S5a). In addition, the regression of the sand content from core ZM01 against the annual mean TC peak intensity over the northwestern Pacific also has a significant and positive correlation (*R* = 0.71, *P* < 0.001, *N* = 35) during the same time interval.

Since the TCs of the other three clusters account for about 66% of the TCs in the northwestern Pacific, it is not surprising that the sand content from core ZM01 and TC frequency affecting the Zhejiang coast in Cluster 1 cannot reflect the variation in the overall TC frequency over the northwestern Pacific (Fig. S5b). Further, our record has a relatively low sedimentation rate (i.e., decadal resolution), so it is hard to judge with certainty whether TC intensity or frequency is causing the observed sand content changes over the past 2000 years. Taking into account the potential influences of the aforementioned factors, we conclude that sand content variations near our site are mainly attributed to variations in TC activity (i.e. intensity and/or frequency of intense TCs) in Cluster 1 of the northwestern Pacific.

We further explored the physical processes responsible for the TC-induced sedimentation using numerical model results (Fig. S6). The mean grain size of surficial sediments surrounding core ZM01 is around 6 μm, which has critical shear stress for resuspension of 0.08 N/m^2^ (1 N/m² = 1 Pa). Very fine sand with a mean grain size of about 63 μm has critical shear stress for resuspension of 0.12 N/m^2^ ^50^. The significant wave height and peak period are respectively 1.9 m and 7.8 s during fair weather. The tidal currents are generally low (i.e., 0.19 m/s) and cause little sediment resuspension with an average wave and current-induced bed shear stress of 0.03 N/m^2^. The significant wave height and peak wave period during Typhoon Chan-hom were 7.6 m and 14.3 s, with a mean current velocity of 0.47 m/s. Storm-induced waves and currents enhanced bed shear stress by more than seven times and caused significant sediment resuspension with an average combined wave-current bed shear stress of 0.26 N/m^2^ (i.e., equivalent to the critical shear stress for the movement of coarse sands with a mean grain size of ~500 μm). These results indicate that the storm can cause resuspension of fine-grained materials, leaving a residue of coarse-grained materials. This mechanism is consistent with previous observations and model studies^51,52^, and therefore supports our interpretation that the sand content represents local intense TC activity. In addition, some of the coarse material may be advected, presumably from the open shelf and/or nearshore, as shown by the bottom current or residual current field during Typhoon Chan-hom (Fig. S7).

### TC activity in eastern China over the past 2000 years

**Fig. 3:**
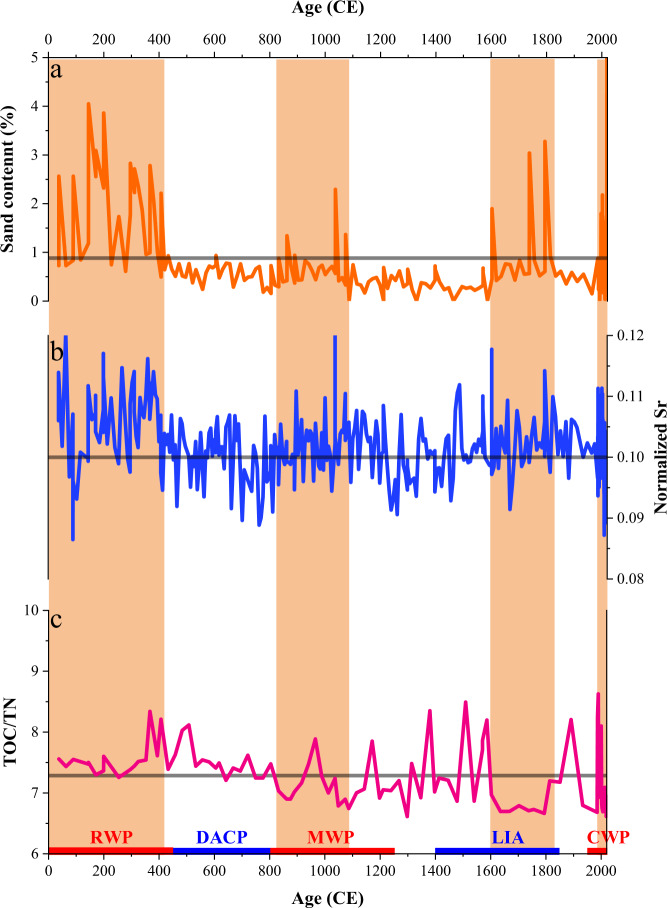
Grain size and geochemical records of core ZM01 from the ZFMB. **a** Sand content, **b** Normalized Sr (normalized to total XRF counts), and **c** TOC/TN ratio. The gray line represents the average value of each parameter. The orange-filled columns represent periods of increased TC activity. The Sr content does not always match the sand content, which is most likely due to the narrower window of the Avaatech XRF (Fig. S1) compared to grain size samples.

Large fluctuations of sand content suggest substantial TC activity changes at the study site over the last two millennia. High sand content occurred during approximately 0–420 CE, 830–1090 CE, 1600–1830 CE, and 1980–2018 CE, while low content occurred during about 420–830 CE, 1090–1600 CE, and 1830–1980 CE (Fig. 3a). Overall, the record indicates that intense TC activity has varied on multi-centennial timescales during this interval. The variations in sand content, total organic matter (TOC)/total nitrogen (TN) ratio, and Sr content exhibit similar patterns, with higher sand content corresponding to decreased TOC/TN and elevated Sr values (Fig. 3b, c). The storm-generated sand layers have higher contents of marine organic matter with lower TOC/TN ratios than background deposits^53^. Sr is found in high concentrations within seaward-sourced, coarse-grained material, representing the contribution of coarser biological debris to sediments^18^.

## Discussion

### TC activity over the last two millennia

**Fig. 4:**
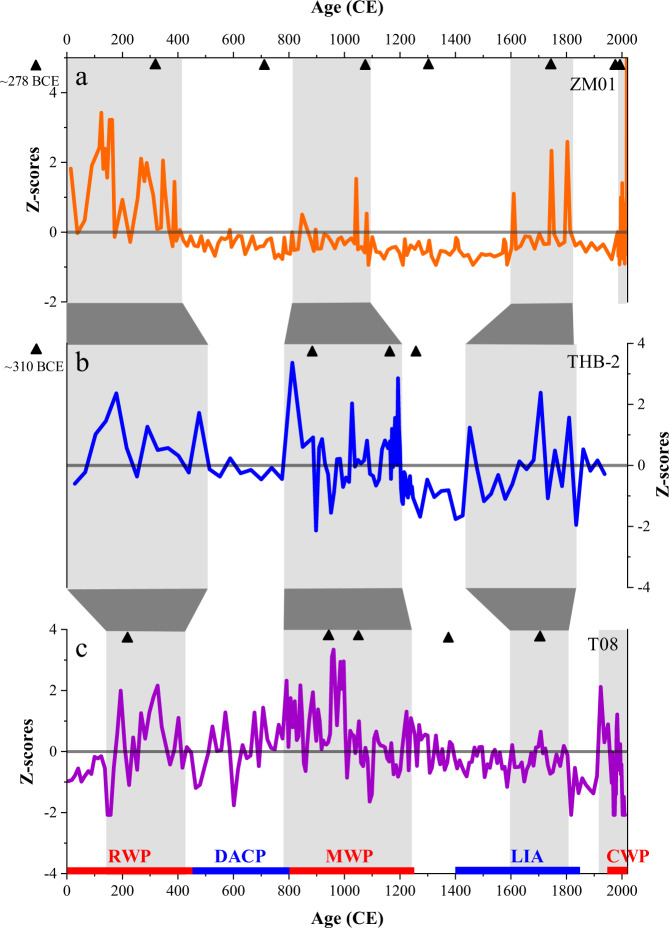
Tropical cyclone records from the ZFMB, offshore eastern China. **a**–**c** Sand content from cores ZM01, THB-2^38^, and T08^25^. All time series are normalized to standard *Z*-scores. Standard *Z*-scores are calculated according to *Z* = (*X* − *V*)/SD; here *X* is the original value, *V* is the averaged value of the time series, and SD is the standard deviation of the time series. In all records, black triangles mark the radiocarbon dates.

**Fig. 5:**
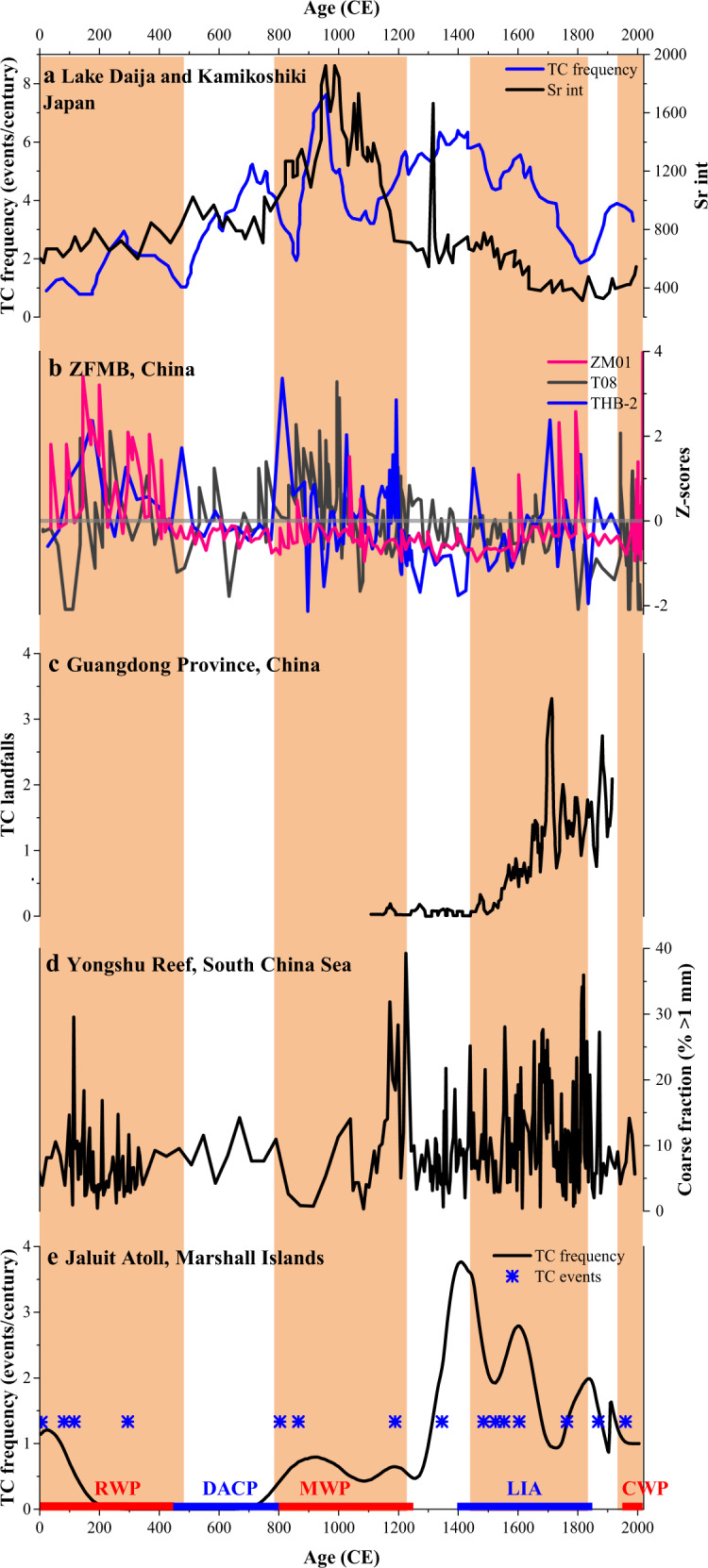
Tropical cyclone reconstructions for the northwestern Pacific. **a** TC frequency reconstructions from Lake Daija (events/century)^55^ and Kamikoshiki (Sr proxy)^18^, Japan. **b** Sand content from cores ZM01, T08^25^, and THB-2^38^ in the ZFMB. **c** Documented TC landfalls in Guangdong Province^27^. **d** Coarse-grained fractions of large wave deposits at Yongshu Reef, South China Sea^54^. **e** Centennial frequency of identified storm deposits from Jaluit Atoll, Marshall Islands^30^.

Our record shows a centennial-scale variability of intense TC activity at the study site over the past 2000 years. Both our record and cores T08^25^ (22 km to the south) and THB-2^38^ (350 km to the south) in the ZFMB show similar changes in sand content, with some minor differences, potentially caused by storm proximity^46–48^, different core sampling resolutions and chronological uncertainties (Fig. 4), and are therefore considered regionally representative. Several periods of increased TC activity are recognized from cores T08, i.e., ~140–440 CE, 830–1230 CE, 1570–1800 CE and 1940–2011 CE, and THB-2, i.e., ~0–480 CE, 790–1200 CE, and 1450–1840 CE. Based on these three independent records, we infer higher TC activity in the ZFMB approximately during the intervals 0–480 CE, 790–1230 CE, and 1940–2018 CE within chronological uncertainty. Our reconstruction of TC activity shows centennial variability similar to other reconstructions from the northwestern Pacific. The TC activity pattern here is almost synchronous with the frequency of coarse deposits at Jaluit Atoll, Marshall Islands^30^, which is close to the cyclogenesis region (Fig. 5e). Previous studies show strong evidence for a seesaw pattern of TC frequency between southern China and Japan^18,20^. However, TC reconstructions for the ZFMB are not exactly consistent with these two regions over the last two millennia (Fig. 5). This partial inconsistency suggests that our reconstruction site is located in the transition zone between the northwestwards (Cluster 1) and westwards (Cluster 2) storms, and may be able to record TC activity in both TC tracks.

Interestingly, our records show an interval of higher TC activity during the LIA (i.e., ~1450–1840 CE). The LIA peak of TC activity in core T08 is less pronounced than in core ZM01, probably due to weaker winds near this core and corresponding lower bed shear stresses caused by the more westerly storm track during that period (Fig. S7). High TC activity in the ZFMB during the LIA is synchronous with the substantial increase in the frequency of coarse deposits at Jaluit Atoll^30^ and Yongshu Reef in the South China Sea^54^, and the enhanced landfall frequency in Guangdong Province^27^ and Japan^18,55^ (Fig. 5). Sedimentary reconstructions in the ZFMB and Japan show higher TC activity during the MWP than during the LIA. However, the LIA peaks in Jaluit Atoll, Yongshu Reef, and Guangdong Province indicate enhanced TC activity relative to the MWP. In addition, TC reconstructions from the ZFMB, Yongshu Reef, and Guangdong Province all show a gradual increase in TC frequency during the LIA, but those from Jaluit Atoll and Japan recorded a gradual decrease.

### Forcing mechanisms and the influence of dust emissions

**Fig. 6:**
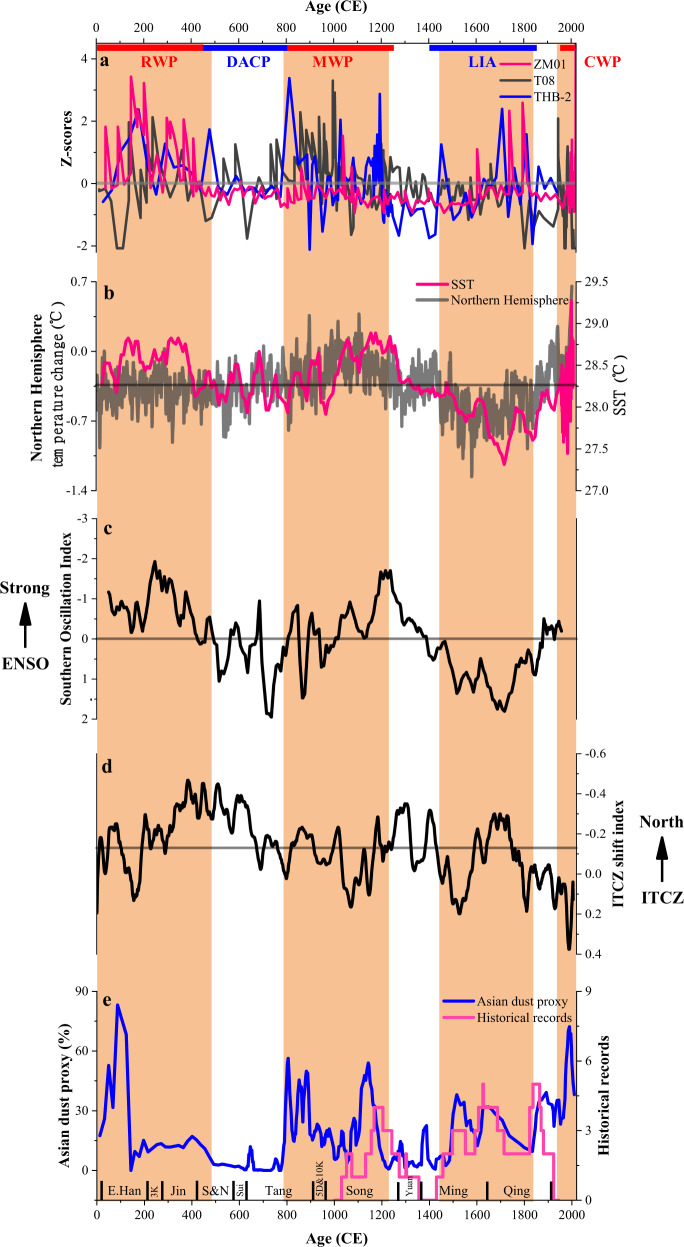
Comparison of TC activity reconstructions with other paleoclimatic proxies in the northwestern Pacific. **a** Normalized sand content from cores ZM01, T08^25^, and THB-2^38^. **b** Temperature reconstructions from the Indo-Pacific warm pool^59^(tropical SST) and Northern Hemisphere^60^. **c** ENSO reconstructions from the equatorial Pacific^35^. **d** Reconstruction of the ITCZ from Klang Cave in southern Thailand, central Indo-Pacific^63^. **e** Later Holocene change in Asian dust activity from Lake Gonghai, northern China^66^, and historical records^67^. Gray lines represent the average value of the parameters, and the orange-filled columns represent periods of increased TC activity. Chinese dynasties since the East Han (E. Han) are indicated at the bottom. 3 K three Kingdoms; S&N Southern and Northern Dynasties, 5D&10 K five Dynasties and ten Kingdoms.

Observation and modeling suggest that intense TC activity should increase with increasing tropical SST^1,10^. In addition, variations in the El Niño–Southern Oscillation (ENSO) and the Intertropical Convergence Zone (ITCZ) also play a key role in governing northwestern Pacific TC activity^30,56–58^. To understand the possible forcing mechanisms related to the variability of past TC activity, we investigated the influence of large-scale oceanic and atmospheric factors (e.g., SST, ENSO, and ITCZ) on TC activity (Fig. 6). Our record of TC activity, together with temperature reconstructions from the Indo-Pacific warm pool and Northern Hemisphere^59,60^ (Fig. 6b), show strong evidence for tropical SST controlling TC activity, with increased TC activity during relatively warm periods (e.g., RWP, MWP, and CWP), and decreased TC activity during relatively cold periods (e.g., DACP). Warm SST anomalies accompanied by tropical tropopause cooling lead to the increase in thermodynamic disequilibrium and thermodynamic efficiency of potential TCs^61^. In combination with reduced vertical wind shear and increased mid-tropospheric moisture content, these conditions potentially result in stronger TCs in warm periods than in cold periods, which is consistent with the conclusions of earlier studies^1,25^.

However, the tropical SST mechanism is difficult to reconcile with the enhanced TC activity during the LIA inferred from our record and other reconstructions from the northwestern Pacific (Fig. 5), as the tropical SST experienced a continuous decline during this interval. Such a shift in TC activity during the LIA challenges the view that lower TC activity is always associated with cold climates. Studies using instrumental observations indicate that ENSO strongly affects the inter-annual variability of TC activity in the northwestern Pacific^3,56^. In the instrumental record, the mean location of TC genesis in the northwestern Pacific shifts to the southeast during El Niño events and TCs tend to recurve north more than in non-event years^56^, which may result in more frequent TCs near our site^4^. A sedimentary reconstruction of the ENSO index from the South China Sea^35^ (Fig. 6c) demonstrates that, in addition to the LIA, variations in ENSO over the past two millennia could have contributed to the variability in our reconstruction. Stronger El Niño events during the MWP may have resulted in more frequent TCs near our site, Jaluit Atoll^30^ and Japan^18,55^, but fewer storm tracks crossing the South China Sea near Yongshu Reef^54^. However, the enhanced magnitude of El Niño events during the RWP cannot explain the increase and decrease of TC frequency near Yongshu Reef and Japan, respectively (Fig. 5a, d). The weakened ENSO causes TC formation to shift westward and TC tracks tending northwestward^4^, which may result in fewer TCs near our site and Jaluit Atoll, in contrast to evidence of high TC activity in our reconstruction during the LIA. Thus, it is likely that other factors have contributed substantially to TC variability over the last two millennia. The north-south migrations of the ITCZ drive hydroclimate variability and may have influenced cyclogenesis across the northwestern Pacific. A northerly ITCZ position causes an eastward shift in cyclogenesis, allowing storms to move more northward^30,62^. The reconstructed ITCZ index^63^ (Fig. 6d) shows a southward displacement during the RWP, causing the storm paths to move northwestward, which may have resulted in more TCs near Yongshu Reef (Fig. 5d), but fewer storm tracks crossing Japan (Fig. 5a). The relative northward displacement of the ITCZ during the LIA may have resulted in few TCs near Yongshu Reef and Guangdong Province, inconsistent with TC reconstructions in these two areas (Fig. 5c, d). Additionally, the results of the Last Millennium experiment in a climate model ensemble may have failed to capture additional LIA cyclogenesis variability associated with ITCZ shifts^30^. Previous studies also suggest that weak EASM during the LIA (Fig. S3) may have shifted cyclogenesis and TC tracks westward^20^, resulting in few TCs in Japan, contrary to the evidence of high TC activity in that region (Fig. 5a). Thus, the widespread increase in TC activity during the LIA in all records of Fig. 5 cannot be explained merely by changes in ENSO, ITCZ, and/or EASM conditions.

This new finding raises the question of whether there is another key factor that increases TC activity during the LIA. Recently, it was hypothesized that dust activity represents one of the significant factors influencing TC activity, suggesting that substantial increases in African dust over the last 200 years and in the early Holocene are coeval with higher Atlantic TC activity, but are admittedly anti-correlated at other times over the last 3000 years^33^. For the Arabian Sea, recent research shows that increased emissions of black carbon and other aerosols have altered the atmospheric circulation in the pre-monsoon season, leading to an increase in the intensity of TCs during that season^64^. However, the influence of Asian dust emissions, mainly transported by EAWM and/or westerly circulation^65^, on TC activity in the northwestern Pacific has not been explored over a longer time window. To unravel the relative importance of Asian dust emissions on TC activity, we compared the proxy time series of dust activity from northern China^66^ and Chinese historical records^67^ (Fig. 6e) with our reconstructed TC activity. One of the main results is that the main phases of increased TC activity correspond well to the increased Asian dust supply over the last 2000 years and vice versa, suggesting a strong response of TC activity to changes in Asian dust emissions. These results suggest that both aerosols and tropical SST are important to the variability of intense TC activity near our site.

Dust storms in northern China are caused by wind erosion of surface soil, which are closely related to both climatic factors (e.g., surface wind speed and precipitation) and to human activities^66^. Asian dust concentrations in the TC genesis region over the northwestern Pacific (Fig. 1a) are mainly controlled by the amount of sediment available for eolian transport in the dust source area and the intensity of EAWM and/or westerly circulation. An ensemble of global aerosol model simulations suggests that dust from East Asia and the Middle East–Central Asia accounts for ~65% of the total dust deposition of the northwestern Pacific^68^, which becomes one of the background atmospheric components affecting TC activity prior to deposition. A dust storm can be intensified by strong winds and grassland degradation in response to reduced rainfall and expanded cultivation in the dust source region. A marked increase in dust storm activity coincided with enhanced EAWM and increased population size (Fig. S8), which generates an increasing amount of sediment available for eolian transport. The dense populations in the dust source region prompted the expansion of cultivation and grassland degradation^66^, resulting in an abrupt increase in dust storm activity. In terms of timing, intensified human activities show strong temporal consistency with the unified dynasties of the Han, Tang, Song, Ming, and Qing dynasties (bottom bar in Fig. 6).

The increased dust emissions from long-distance transport to areas where TCs are generated and developed may have further enhanced TC activity when the SST was high (i.e., RWP, MWP, and CWP). In contrast, periods of decreased TC activity are coeval with periods of decreased dust storm activity and declining population in the dust source region, corresponding to times of weaker EAWM intensity (i.e., DACP and 1230–1450 CE). During the early phase of RWP, when dust emissions were high, TC activity was also exceptionally high. During the later phase of RWP, the SST was slightly higher, but dust emissions were reduced, TC activity was also lower. A pronounced increase in Asian dust activity occurred during the LIA, together with enhanced EAWM and increased population. The resulting high aerosol concentrations in the region of TC genesis may have contributed to the enhanced TC activity by releasing more latent heat and promoting the development of convection^69,70^, even though the SST reached its lowest value over the past 2000 years. This finding suggests that increased Asian dust activity was likely a major factor controlling the widespread increase in TC activity in the northwestern Pacific during the LIA.

This study emphasizes the importance of Asian dust emissions on TC activity, especially when SST is below a particular threshold. Further modeling studies of the impact of Asian dust emissions on TC activity under a wide range of environmental conditions (e.g., SST and wind shear) are needed to supplement our findings. In the future, if the intensified human activity in northern China continues, coupled with projected enhanced EAWM^71^, more Asian dust will be supplied to the TC region in the northwestern Pacific. Combined with continued global warming, this dust supply has the potential to cause more severe TCs in eastern China.

## Methods

### Sediment archives and analytical methods

A sediment core was collected from the ZFMB using a gravity corer in May 2018 (Fig. 1). Core ZM01 (508 cm long; 57 m water depth; 28.69°N, 122.41°E) was chosen to maximize the TC record, in a shallow inner-shelf (insubstantial tsunami impacts) and away from the Changjiang estuary (less affected by river floods). Elemental concentrations (e.g., Sr) were determined by using an Avaatech X-ray fluorescence core scanner (XRF) at 0.5 cm intervals. Before scanning, the split-core surfaces were first flattened and covered with a thin (4 μm) ultralene film to avoid contaminating the measurement prism of the core scanner. To ensure signal stability during analytical runs, four powdered standards were analyzed before and after analysis of the sediment core to monitor signal drift. The grain size was measured on sub-samples at 1 cm intervals using a laser Malvern Mastersizer 2000 with a duplicate measurement error <3%. Preservation of sandy laminae indicates that the core is not heavily bioturbated, which is supported by other studies from the same region, e.g., cores C0702^72^ (58 km to the north) and T08^25^. The results show that most of the calcareous and siliceous biological debris are derived from TC events, but partly from redistributed by bioturbation. Considering the points above, the biological debris was preserved when preparing sediments to present the TC event more clearly, without using HCl and NaOH to remove carbonate and diatoms. The bulk sediments were dispersed by adding 0.05 mol/L (NaPO_3_)_6_ solution (10–20 mL) and by ultrasonic treatment for 15 s before grain size measurement. In this study, we used the grain size-standard deviation approach to extract the TC-sensitive grain-size fraction.

Total organic carbon (TOC) and total nitrogen (TN) contents were measured with an Elementar Vario PYRO Cube elemental analyzer at 2 cm intervals. Approximately 0.5 g of freeze-dried and grounded sediment was treated with about 3 mL of 1 M HCl for 16 h to remove inorganic carbon and the residue was washed to neutrality and then freeze-dried for 24 h. Around 60 mg of decarbonated sediment was crimp sealed in a tin capsule. The long-term analytical precision determined on repeated analyses of samples and standards is ±0.1% for TOC and TN. Clay minerals were identified by X-ray diffraction (XRD) using a PANalytical X’Pert PRO diffractometer at 2 cm intervals on oriented mounts of decarbonated clay-sized (<2 μm) particles. The preparation of oriented mounts followed the method described in detail by Liu et al. (2004)^73^. Each sample was measured three times under the conditions of air-drying, ethylene-glycol solvation for 24 h, and heating at 490 °C for 2 h. The clay minerals were identified and interpreted mainly on the basis of the (001) basal reflections on the three XRD diagrams. The proportions of clay minerals were calculated semi-quantitatively using the MacDiff software, based on the peak areas of basal reflections of smectite (17 Å), illite (10 Å), and kaolinite/chlorite (7 Å) on the glycolated curve. Relative proportions of kaolinite and chlorite were determined based on the ratio of the 3.57/3.54 Å peak areas. The accuracy of the semi-quantitative evaluation of each clay mineral based on the XRD method is ~5%. Clay minerals (e.g., illite/smectite and kaolinite/chlorite ratios) contain valuable information on sediment provenance in the ZFMB, East China Sea^74^.

Two isotopic dating methods were used to establish age control. The activity of ^210^Pb, ^137^Cs, and ^226^Ra within sediments was measured using HPGe γ spectrometry (Canberra GSW275L), to provide an age model and its uncertainty (±2 years) for the top of the core based on the constant initial concentration method^75^. ^210^Pb_ex_ activity was calculated by subtracting ^226^Ra activity (i.e., the ^210^Pb background value) from the total ^210^Pb activity. The ^137^Cs-based ages were determined based on the key time markers, e.g., 1963 CE time marker (i.e., the peak in thermonuclear weapons testing coincided with the onset of a moratorium on such tests in 1963 CE). Centennial-to-millennial scale chronologies were constrained by ^14^C-Accelerator Mass Spectrometry (^14^C-AMS) radiocarbon dates of benthic foraminifera (Table S1). Radiocarbon dates were calibrated to calendar ages using the CALIB 8.2, with a marine reservoir correction of 500 years (Δ*R* = 96 ± 46 is based on the apparent ^14^C ages of the bivalve shells)^76,77^. Age model and associated 95% uncertainties were computed using the Undatable program^78^. Throughout the record, we assume event beds are deposited in days. Thus, we condense all event beds down to 1 cm, remove them for age-depth modeling, and then reinsert them following age-depth estimation. We distinguished event beds from background variations in sand content by following the methods of Lane et al. (2011)^79^ and Wallace et al. (2021)^47^. We calculated the coarse anomaly by removing a ten-point moving average from the sand content data. Event beds are coarse anomaly peaks that exceed 80% of the cumulative distribution function of the coarse anomaly data (the threshold for core ZM01 is 0.17%). Based on this criterion we identify 35 event beds in core ZM01 (Fig. S2). Annual maximum wind speed data of TCs around the core site (120–124°E, 26–30°N; Fig. 1b) are calculated from the Climate Forecast System with a spatial resolution of 0.2° × 0.2° in 2011–2018 CE (0.3° × 0.3°in 1984–2010 CE) and temporal resolution of 1 h. The number of TC events affecting the Zhejiang coast (wind speed >10.8 m/s) was calculated from the tropical cyclone database of the China Meteorological Administration (www.typhoon.gov.cn).

### Model and simulation

The hydrodynamic conditions (i.e., waves and currents) of the bottom boundary layer (at a height of 1 m above the seabed) were reconstructed by using the finite-volume community ocean model (FCVOM) with a horizontal resolution of 0.5 km for the coastal zone. The high-resolution bathymetry data for the Zhejiang coast and the Changjiang estuary were provided by the Ocean and Fisheries Bureau of Zhejiang Province. Wind data is the reanalysis data provided by the National Centers for Environmental Prediction (NCEP) with a temporal and spatial resolution of 6 h and 0.25°, respectively. The external forcing for the present model includes tide, wind speed, air pressure, and river runoff. The model was evaluated by field observations of storm surge and significant wave height caused by Typhoon Chan-hom, allowing us to estimate the hydrodynamic conditions in both fair weather and during storms, and whether the bottom sediment from the ZFMB can be resuspended. The values of the model skill are typically 0.94^80^, indicating the high performance of the model. The models were operated from June 30, 2015 to July 14, 2015, which covered the period of Typhoon Chan-hom. In addition, the effects of aerosol on the intensity of an idealized TC were investigated by coupling an explicit treatment of both aerosol activation and electrification parameterizations with the Weather Research and Forecasting Model (WRF; version 3.4.1)^69^. The WRF V3.4.1 mesoscale model uses clean and polluted aerosol scenarios^81^ to simulate the evolution of an idealized TC by using the Morrison microphysical scheme. The entire simulation duration was 144 h, with a horizontal resolution of 15 km.

## Supplementary information

Supplementary Information

## Data Availability

The sand content data are available at: 10.6084/m9.figshare.18316556.v1.
