# Author Correction: Northwestern Pacific tropical cyclone activity enhanced by increased Asian dust emissions during the Little Ice Age

**DOI:** 10.1038/s41467-022-30171-4

**Published:** 2022-05-05

**Authors:** Yang Yang, David J. W. Piper, Min Xu, Jianhua Gao, Jianjun Jia, Alexandre Normandeau, Dongdong Chu, Liang Zhou, Ya Ping Wang, Shu Gao

**Affiliations:** 1 School of Marine Science and Engineering, Nanjing Normal University, Nanjing, 210046 China; 2 Natural Resources Canada, Geological Survey of Canada (Atlantic), Bedford Institute of Oceanography, Dartmouth, NS B2Y 4A2 Canada; 3 School of Geography and Ocean Science, Ministry of Education Key Laboratory for Coast and Island Development, Nanjing University, Nanjing, 210093 China; 4 State Key Laboratory for Estuarine and Coastal Research, School of Marine Sciences, East China Normal University, Shanghai, 200062 China; 5 Institute of Physical Oceanography and Remote Sensing, Ocean College, Zhejiang University, Zhoushan, 316000 China; 6 School of Geography, Geomatics and Planning, Jiangsu Normal University, Xuzhou, 221116 China

**Keywords:** Palaeoceanography, Palaeoclimate

Correction to: *Nature Communications* 10.1038/s41467-022-29386-2, published online 31 March 2022.

In this article the affiliation details for Dongdong Chu were incorrectly given as ‘Changjiang River Scientific Research Institute, Wuhan 430010, China.’ but should have been ‘Institute of Physical Oceanography and Remote Sensing, Ocean College, Zhejiang University, Zhoushan, 316000, China’. The original article has been corrected.

