# Supplementary material for: Northwestern Pacific tropical cyclone activity enhanced by increased Asian dust emissions during the Little Ice Age
Source: Nat Commun. 2022 Mar 31;13:1712. doi: 10.1038/s41467-022-29386-2 (PMC8971422; doi:10.1038/s41467-022-29386-2)
Supplement: Supplementary file 1 — Supplementary Information [file 41467_2022_29386_MOESM1_ESM.pdf]

Supplementary Information

Northwestern Pacific tropical cyclone activity enhanced by increased Asian dust emissions during the Little Ice Age

Yang et al.

High sedimentation rate in the last century

Depositional environment changes through time may potentially affect the stability of the sedimentary record, such as sea-level changes, hydrodynamic conditions, sediment supply, and shoreline position[1-2]. The relative sea level along the Zhejiang coast has remained stable over the past 2,000 years[3], fluctuating within a range of less than 2 m. Numerical simulations show that the late-Holocene paleo-tidal regime is similar to the present-day current field[4]. The sediments of the Zhejiang mud belt are derived mainly from the Changjiang River, and to a lesser extent from local small mainland rivers[5-6]. Previous studies have suggested that the Changjiang River has provided a steady supply of sediment to the Zhejiang mud belt over the past 2,000 years[6-7], as indicated by a constant deposition of clay minerals in this study (Fig.S1). In addition, a local shoreline reconstruction with the same geological background conditions shows no significant changes over the past 2,000 years[3]. Therefore, changes in the depositional environment during this interval appears insufficient to substantially alter the stability of core ZM01 to record tropical cyclone (TC) events. However, based on age-models that use ^{210}Pb-^{137}Cs and radiocarbon dating as age-control, the sedimentation rate of the lower part (192-40 cm) of core ZM01 is ~ 0.80 mm/year, but is ~12.4 mm/year in the upper 40 cm (~ last 100 years). The upper 40 cm spanning the observational period has an order of magnitude higher resolution than the rest of the record, potentially providing an important means to assess the role of both TC frequency and intensity in creating storm event layers. Considering the potential influence of the above factors, we infer that the processes governing the formation and preservation of storm event layers are likely to remain stable throughout the record.

For the Zhejiang coast, human activities affect the sedimentation rate in coastal areas by influencing the sediment flux to the sea from local small rivers (e.g., Jiaojiang and Oujiang rivers; Fig.1). According to Xue et al. (2018)[5] and Jia et al. (2018)[8], the sediment

input from the small rivers within the Zhejiang mud belt was estimated to be about 15 Mt/year (i.e., million tons per year) during the last century, accounting for about 4% of the total sediment deposition flux within this region (~ 350 Mt/yr). Therefore, coastal development in the Zhejiang coastal areas during the last century did not significantly change the sedimentation rate near our site. The Changjiang River is the strongest driver for the sedimentation within the Zhejiang mud belt, with a mean contribution of ~75% during the last century[5,8]. With increasing population in the Changjiang catchment, soil erosion was intensified, and the sediment load towards the sea gradually increased during the last century[9]. Therefore, the rapid increase in sedimentation rate coincides with the timing of intensive impacts of human activity and rapid economic development in the Changjiang catchment. After 2003 CE, the Changjiang River has experienced a sharp reduction in sediment flux mainly due to dam construction (e.g., the Three Gorges Dam), which led to partial erosion of Changjiang Subaqueous Delta[10]. However, the current sedimentary environment within the Zhejiang mud belt remains relative stable, but is becoming increasingly unstable[10]. The reduction in river-induced sediment supply was partly compensated by eroded sediment from the Changjiang Subaqueous Delta[8], and the response time of this area to the insufficient sediment supply from the Changjiang River is likely to be $>10^1$ years[10].

Numerical simulation of the effect of aerosols on the intensity of an idealized tropical cyclone

We further explored the dynamic mechanism behind the relationship between TC activity and Asian dust emissions by using the modeling outputs from the WRF model[11]. Previous studies have mostly focused on the influence of aerosols on the mature stage of TCs, and suggested that increased aerosols suppressed the TC intensity[11-13]. It was found that TC intensity might be more susceptible to the impacts of aerosols during their developing stages and less in the TC's mature stage[14]. The model results show that the nucleation of aerosols by Asian dust has an effect on TC intensity that is opposite in the developmental and the mature stages of a TC[11-12]. In the developmental stage of a TC, the increase in aerosol concentration results in the formation of more but smaller cloud droplets, which reduce warm rain by suppressing collision and coalescence processes[12]. These small

cloud droplets are transported to the mixed-phase region and freeze to form more ice particles[14]. The freezing process releases more latent heat, promoting the development of convection, which enhances the intensity of the TC. In the mature stage, more ice particles melt into rain, and rain particles hinder the updraft as they fall[15]. The evaporation of rainfall particles on the sea surface absorbs more latent heat, lowers the temperature in the area, inhibits the development of convection, and thereby reduces the TC intensity. The location of the maximum wind speed of the intense TCs making landfall in Zhejiang Province (1949-2019 CE; Fig. S4) demonstrates that the intense TCs affecting the ZFMB are mainly in the late developmental stage. Therefore, the increased Asian dust supply may enhance TC intensity in this region.

Supplementary Figures

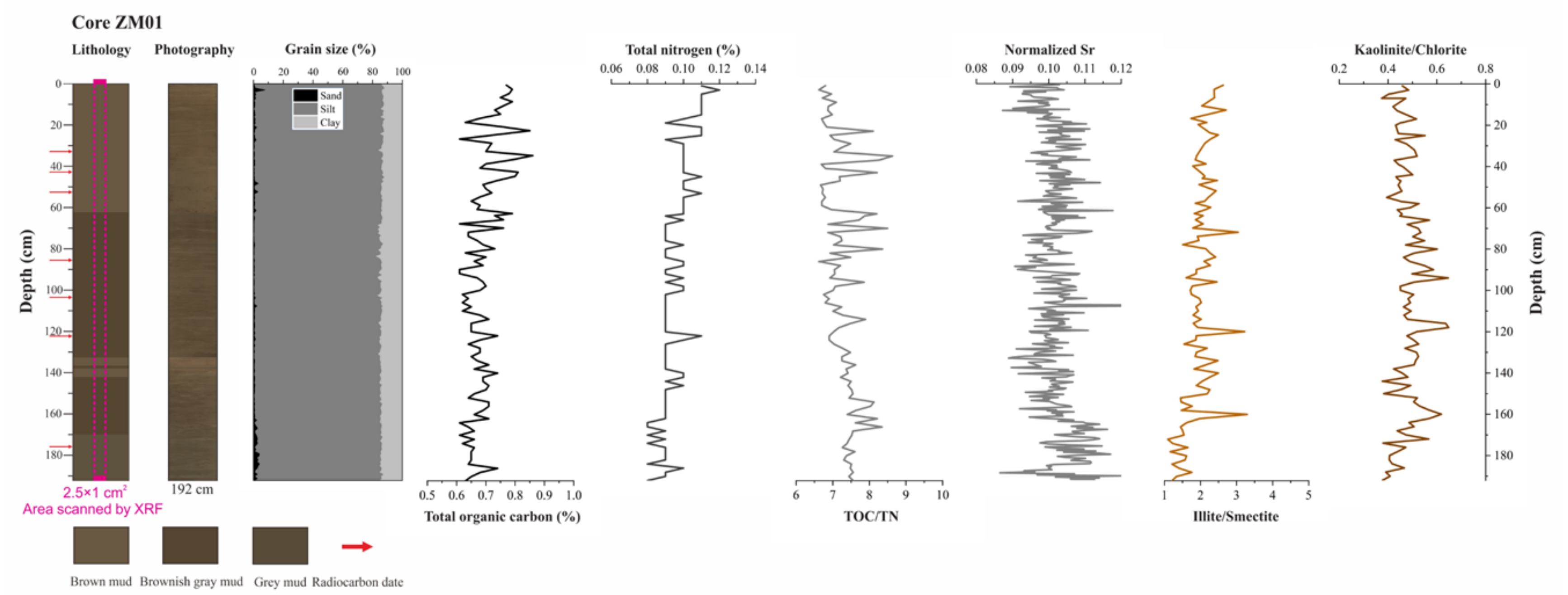

Figure S1. Depth profiles for core ZM01. Lithology, photographs, grain size composition, total organic matter (TOC), total nitrogen (TN), TOC/TN ratio, normalized Sr (normalized to total XRF counts) and mineralogical proxies (illite/smectite and kaolinite/chlorite ratios) of core ZM01.

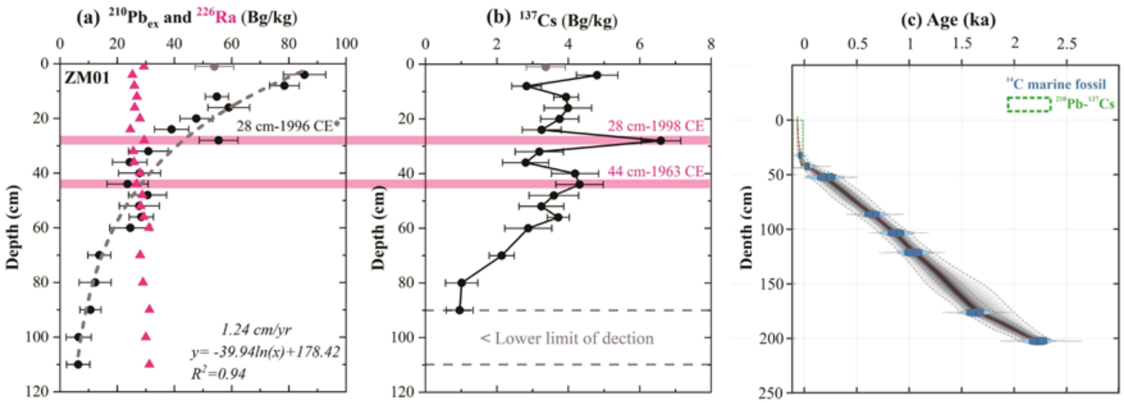

Figure S2. Chronology of core ZM01. (a) Profile of ^{210}Pb-^{226}Ra activity. * represents the age determined by ^{210}Pb dating at 28 cm. (b) Profile of ^{137}Cs activity. The red lines indicate the 1998 CE and 1963 CE ^{137}Cs time markers. (c) Age-depth model from *Undatable* program using ^{14}C-AMS dates. *Undatable* results show the single best model for each depth (red line) and the 95.4% and 68.2% confidence intervals (grey dotted line). Based on the event bed threshold, we identify 36 event beds in core ZM01, i.e., 3, 5, 7, 17, 19, 21, 27, 37, 48, 52, 61, 64-65, 77-78, 85, 92, 103, 107, 120, 123, 126, 129, 145, 162, 166, 170, 173, 178-179, 181, 183-185, 188 and 191 cm. The top 1 cm sediments of core ZM01 may have been subjected to vertical and horizontal material mixing because higher ^{210}Pbex activity occurs at > 1 cm of the core, and therefore was not included for analysis.

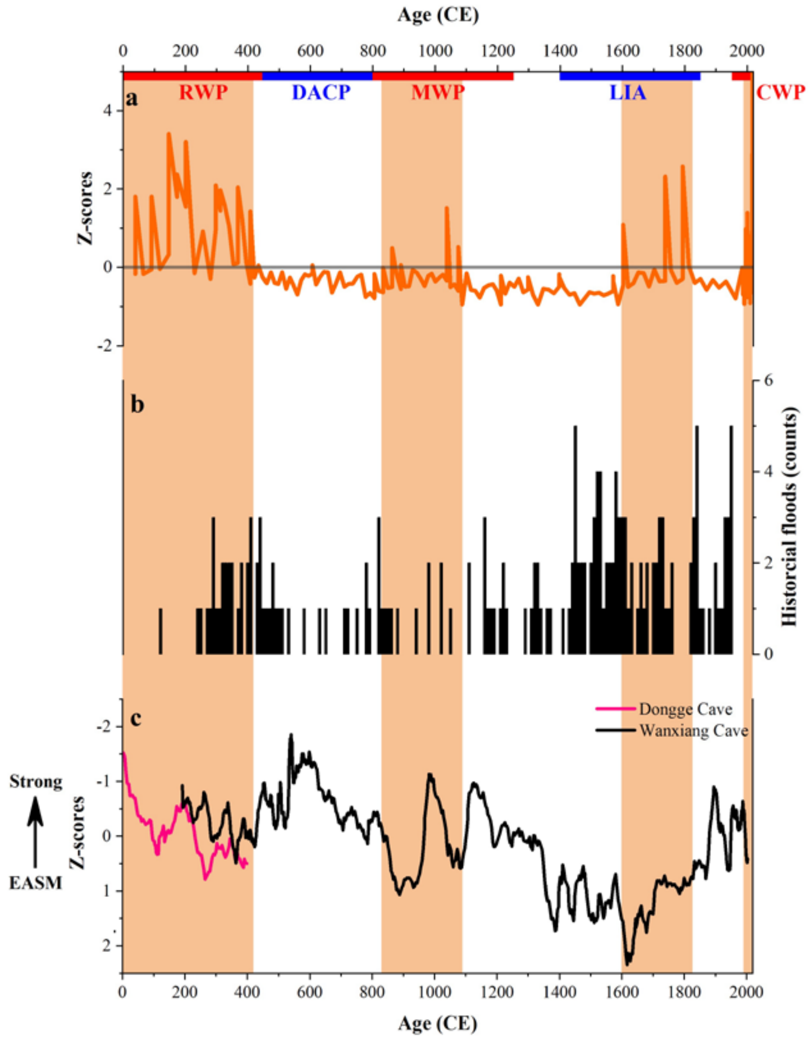

Figure S3. Comparison of TC reconstruction with other paleoclimatic proxies. (a) Sand content from core ZM01. (b) Historical flood records in the Nanjing Reach, Changjiang River[16]. (c) East Asian Summer Monsoon (EASM) intensity from Dongge Cave[16], southern China and WanXiang Cave[18], central China.

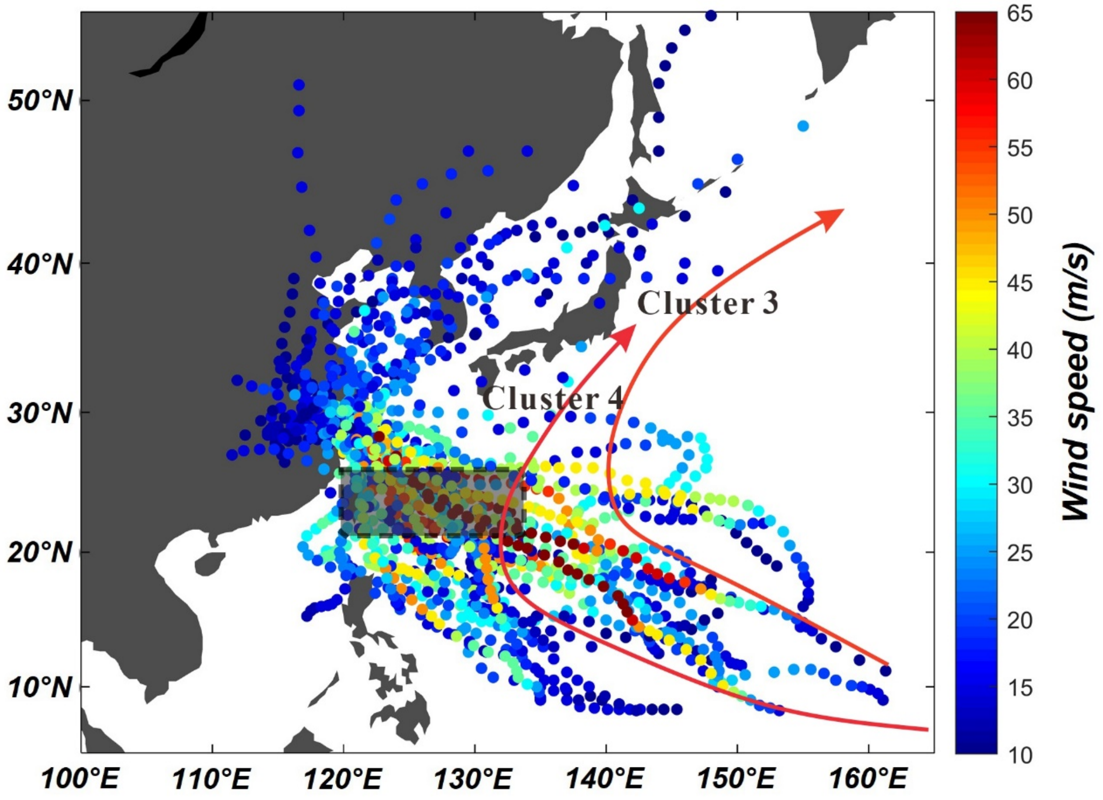

Figure S4. Information on the track and intensity of the intense TCs making landfall on the Zhejiang coast (1949-2019 CE). The black shaded area shows the average range of the maximum wind speed of the typhoon (i.e., 120–134°E, 21–27°N). Typical tracks of TCs in Clusters 3 and 4 are also shown[19].

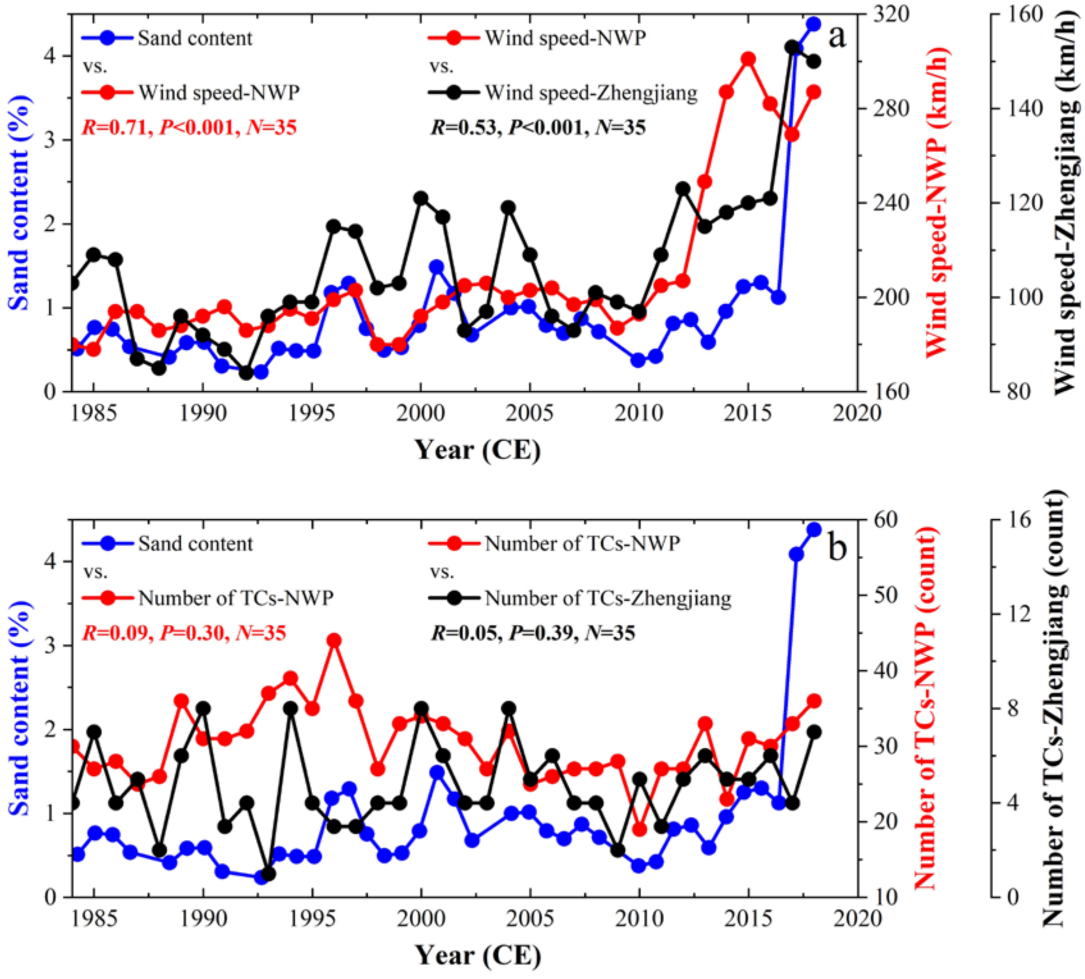

Figure S5. Comparison of the sand content of core ZM01 with TC frequency and intensity during 1984-2018 CE. (a) Comparison of the annual mean TC peak intensity over the northwestern Pacific with the sand content of core ZM01 and the maximum wind speed of TCs affecting the Zhejiang coast. (b) Comparison of the number of TC events over the northwestern Pacific with the sand content of core ZM01 and TC frequency affecting the Zhejiang coast.

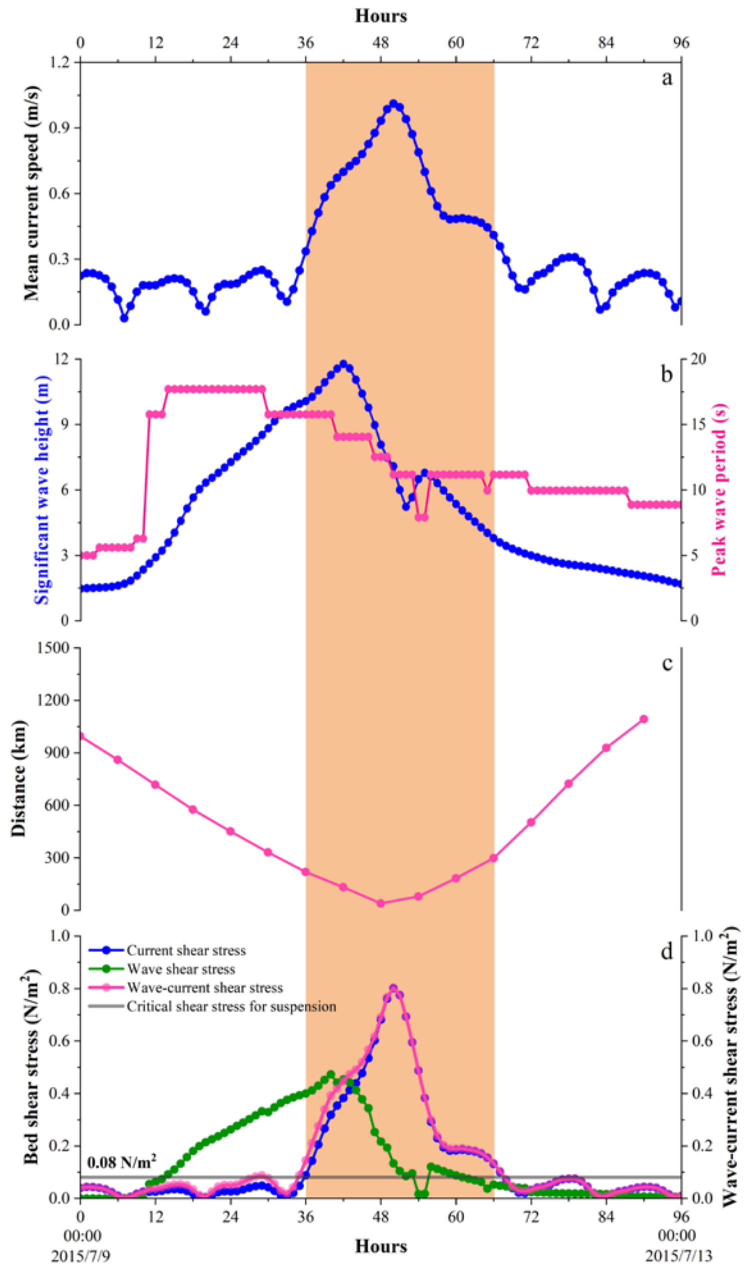

Figure S6. Modeled hydrodynamic conditions of the bottom boundary layer (at a height of 1 m above seabed) during the passage of 2015 CE Typhoon Chan-hom. Time series of (a) mean current speed; (b) significant wave height and peak wave period; (c) the distance between the center of Typhoon Chan-hom and core ZM01; and (d) current, wave, and combined wave- and current-induced bed shear stress. For fine silt of 6 μm, critical shear stress for resuspension = 0.08 N/m^2 and orange bar marks the period with sediment resuspension.

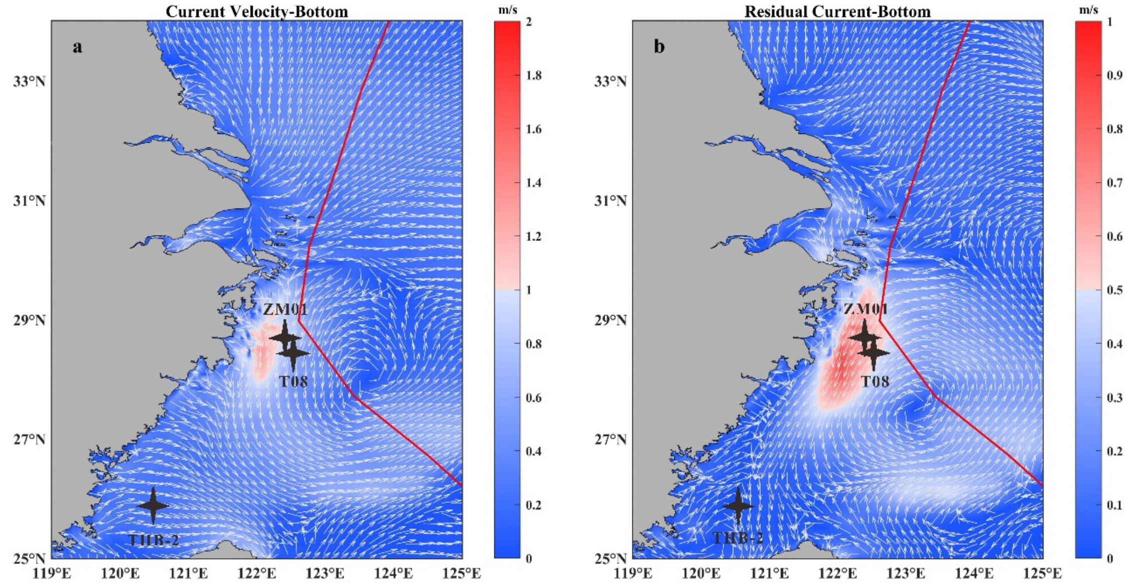

Figure S7. Modeled bottom currents during the passage of 2015 CE Typhoon Chan-hom (red line). (a) Current velocity. (b) Residual current.

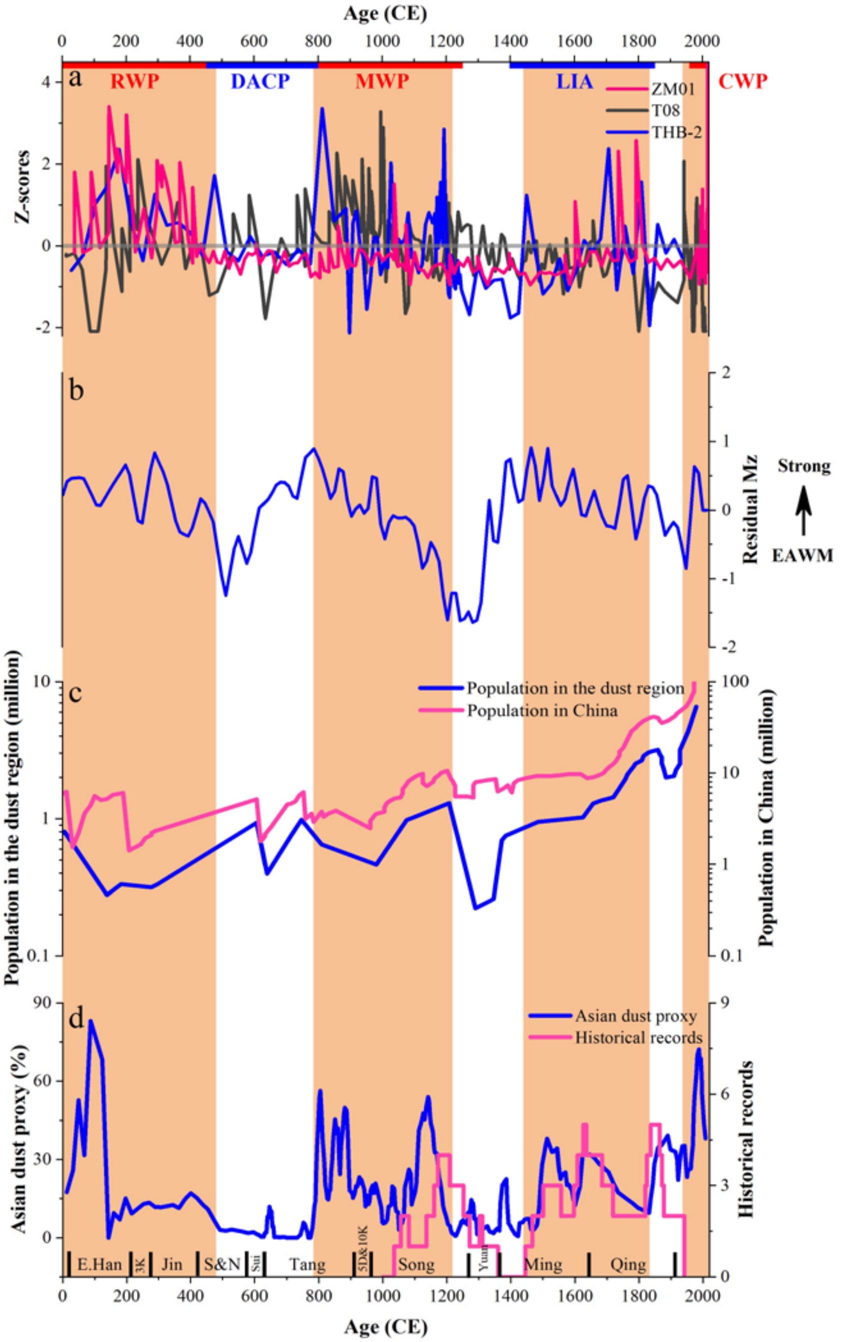

Figure S8. Comparison of TC activity reconstructions with Asian dust activity, population and East Asian Winter Monsoon (EAWM). (a) Normalized sand content from cores ZM01, T08[20] and THB-2[21]. (b) EAWM intensity reconstructions from residual magnetic susceptibility (MS) from the southern Chinese Loess Plateau[22]. (c) Population variations in China and in the dust source region (i.e., Shanxi, Shaanxi, Ningxia, and Inner

Mongolia)[23]. (d) Late Holocene change in Asian dust activity from Lake Gonghai, northern China[24] and historical records[25].

Supplementary Table S1. List of ^{14}C-AMS dates of core ZM01.

Lab ID	Material	Depth (cm)	Δ^{14}C (‰)	Uncorrected ^{14}C age (1σ, yr BP)	Calibrated age (1σ, yr BP)
1	Benthic forams	30-35	-46.1±2.2	310±20[a]	-40±20
2	Benthic forams	41-44	-65.2±2.2	475±20	27±22
3	Benthic forams	50-55	-107.8±3.2	850±30	210±88
4	Benthic forams	85-88	-162.3±3.3	1355±35	646±76
5	Benthic forams	101-106	-187.0±1.8	1595±20	874±79
6	Benthic forams	120-123	-202.3±3.2	1745±35	1039±89
7	Benthic forams	175-178	-256.0±1.8	2310±20	1628±82
8	Benthic forams	200-205	-299.5±2.1	2790±25	2228±86

[a] This date was calibrated to calendar ages using a ΔR value of -150±17 as a strong positive-to-negative shift in ΔR occurred in the Kuroshio region during recent decades[26].

Supplementary References

1. Wallace, E. J., Donnelly, J. P., van Hengstum, P. J., Winkler, T. S., McKeon, K., MacDonald, D., d'Entremont, M., Sullivan, R. M., Woodruff, J.D., Hawkes, A.D., and Maio, C., 2021. 1,050 years of hurricane strikes on Long Island in The Bahamas. Paleoceanography and Paleoclimatology, 36(3), e2020PA004156.
2. Yang, Y., Zhou, L., Normandeau, A., Jia, J.J., Yin, Q.J., Wang, Y. P., Shi, B.W., Gao, L., and Gao, S., 2020. Exploring records of typhoon variability in eastern China over the past 2000 years. Geological Society of America Bulletin, 132(11-12), 2243-2252.
3. Feng, H.Z., and Wang, Z.T., 1986. Zhejiang’s Holocene coastal shift and sea level change. Journal of Hangzhou University, 13(1), 100-107.
4. Uehara, K., and Saito, Y., 2003. Late Quaternary evolution of the Yellow/East China Sea tidal regime and its impacts on sediments dispersal and seafloor morphology. Sedimentary Geology, 162(1-2), 25-38.

5. Xue, C.F., Jia, J.J., Gao, S., Cai, T.L., Yao, H.J., Li, J., Chen, Y.N., and Xia, X.M., 2018. The contribution of middle and small rivers to the distal mud of subaqueous Changjiang Delta: results from Jiaojiang River and Oujiang River. Haiyang Xuebao ,40 (5), 75-89.
6. Wang, H.J., Saito, Y., Zhang, Y., Bi, N.S., Sun, X.X., and Yang, Z.S., 2011. Recent changes of sediment flux to the western Pacific Ocean from major rivers in East and Southeast Asia. Earth-Science Reviews, 108(1-2), 80-100.
7. Bi, L., Yang, S.Y., Zhao, Y., Wang, Z.B., Dou, Y.G., Li, C., Zheng, H.B., 2017. Provenance study of the Holocene sediments in the Changjiang (Yangtze River) estuary and inner shelf of the East China sea. Quaternary International, 441, 147-161.
8. Jia, J.J., Gao, J.H., Cai, T.L., Li, Y., Yang, Y., Wang, Y. P., Xia, X.M., Li, J., Wang, A.J., and Gao, S., 2018. Sediment accumulation and retention of the Changjiang (Yangtze River) subaqueous delta and its distal muds over the last century. Marine Geology, 401, 2-16.
9. Gao, J.H., Xu, X.N., Jia, J.J., Kettner, A.J., Xing, F., Wang, Y.P., Yang, Y., Qi, S.H., Liao, F.Q., Li, J., Bai, F.L., Zou, X.Q., and Gao, S., 2015. A numerical investigation of freshwater and sediment discharge variations of Poyang Lake catchment, China over the last 1000 years. The Holocene, 25(9), 1470-1482.
10. Gao, J. H., Shi, Y., Sheng, H., Kettner, A. J., Yang, Y., Jia, J. J., Wang, Y.P., Li, J., Chen, Y.N., Zou, X.Q., and Gao, S., 2019. Rapid response of the Changjiang (Yangtze) River and East China Sea source-to-sink conveying system to human induced catchment perturbations. Marine Geology, 414, 1-17.
11. Zhao, P.G., Yin, Y., Xiao, H., and Kang, H.Q., 2016. Numerical simulations of the effects of aerosol on the intensity and electrification of tropical cyclone. Journal of the Meteorological Sciences, 36(1), 1-9.
12. Cotton, W. R., Krall, G. M., and Carrió, G. G., 2012. Potential indirect effects of aerosol on tropical cyclone intensity: Convective fluxes and cold-pool activity. Tropical Cyclone research and review, 1(3), 293-306.
13. Zhang, H.N., McFarquhar, G. M., Cotton, W. R., and Deng, Y., 2009. Direct and indirect impacts of Saharan dust acting as cloud condensation nuclei on tropical cyclone eyewall development. Geophysical Research Letters, 36, L06802.

14. Rosenfeld, D., Woodley, W. L., Khain, A., Cotton, W. R., Carrió, G., Ginis, I., and Golden, J. H., 2012. Aerosol effects on microstructure and intensity of tropical cyclones. Bulletin of the American Meteorological Society, 93(7), 987-1001.
15. Tao, W. K., Li, X.W., Khain, A., Matsui, T., Lang, S., and Simpson, J., 2007. Role of atmospheric aerosol concentration on deep convective precipitation: Cloud-resolving model simulations. Journal of Geophysical Research: Atmospheres, 112, D24S18.
16. Bian, G.H., 2008. Meteorological Disasters in China Ceremony—Jiangsu Province (in Chinese). Meteorological Press, Beijing.
17. Wang, Y.J., Cheng, H., Edwards, R.L., He, Y.Q., Kong, X.G., An, Z.S., Wu, J.Y., Kelly, M.J., Dykoski, C.A., and Li, X.D., 2005. The Holocene Asian monsoon: links to solar changes and North Atlantic climate. Science 308, 854-857
18. Zhang, P.Z., Cheng, H., Edwards, R. L., Chen, F.H., Wang, Y.J., Yang, X.L., Liu, J., Tan, M., Wang, X.F., Liu, J.H., An, C.L., Dai, Z.B., Zhou, J., Zhang, D.Z., Jia, J.H., Jin, L.Y., Johnson, K. R., 2008. A test of climate, sun, and culture relationships from an 1810-year Chinese cave record. Science, 322(5903), 940-942.
19. Mei, W., and Xie, S. P., 2016. Intensification of landfalling typhoons over the northwest Pacific since the late 1970s. Nature Geoscience, 9(10), 753-757.
20. Zhou, X., Liu, Z.H., Yan, Q., Zhang, X.L., Yi, L., Yang, W.Q., Xiang, R., He, Y.X., Hu, B.Q., Liu, Y., and Shen, Y.N., 2019. Enhanced tropical cyclone intensity in the western North Pacific during warm periods over the last two millennia. Geophysical Research Letters, 46, 9145-9153.
21. Hu, B.Q., Li, J., Zhao, J.T., Wei, H.L., Yin, X.J., Li, G.G., Liu, Y., Sun, Z.L., Zou, L., Bai, F.L. Dou, Y.G., Wang, L.B., and Sun, R.T., 2014. Late Holocene elemental and isotopic carbon and nitrogen records from the East China Sea inner shelf: Implications for monsoon and upwelling. Marine Chemistry, 162, 60-70.
22. Kang, S.G., Wang, X.L., Roberts, H.M., Duller, G.A., Cheng, P., Lu, Y.C.. and An, Z.S., 2018. Late Holocene anti-phase change in the East Asian summer and winter monsoons. Quaternary Science Reviews, 188, 28-36.
23. Zhao, W. L. and Xie, S. J., 1988. History of Population in China (In Chinese). People's Publishing House.

24. Chen, F.H., Chen, S.Q., Zhang, X., Chen, J.H., Wang, X., Gowan, E.J., Qiang, M.R., Dong, G.H., Wang, Z.L., Li, Y.C., Xu, Q.H., Xu, Y.Y., Smol, J.P., and Liu, J.B., 2020. Asian dust-storm activity dominated by Chinese dynasty changes since 2000 BP. Nature Communications, 11(1), 1-7.
25. Zhang, D.E., 1984. Climatic analysis of the dust storm weather in Chinese history. Science China, 3, 278-288.
26. Hirabayashi, S., Yokoyama, Y., Suzuki, A., Miyairi, Y., and Aze, T., 2017. Short-term fluctuations in regional radiocarbon reservoir age recorded in coral skeletons from the Ryukyu Islands in the north-western Pacific. Journal of Quaternary Science, 32(1), 1-6.
